# A new model for evaluating maintenance energy requirements in dogs: allometric equation from 319 pet dogs

**DOI:** 10.1017/jns.2017.50

**Published:** 2017-10-12

**Authors:** Guilhem Divol, Nathalie Priymenko

**Affiliations:** 1 279, chemin des Antiquailles, 30000 Nîmes, France; 2 TOXALIM, Université de Toulouse, Institut National de la Recherche Agronomique (INRA), École Nationale Vétérinaire de Toulouse (ENVT), 23 chemin des Capelles, BP 87614, 31076 Toulouse cedex, France

**Keywords:** Canine nutrition, Maintenance energy requirements, Allometric equations, Pet dogs, BCS, body condition score, BW, body weight, MER, maintenance energy requirements

## Abstract

Reports concerning maintenance energy requirements (MER) in dogs are common but most of the data cover laboratory or utility dogs. This study establishes those of healthy adult pet dogs and the factors which cause these energy requirements to vary. Within the framework of a nutrition teaching exercise, each student followed a pet from his entourage and gathered accurate records of its feeding habits. Data have been restricted to healthy adult dogs with an ideal body weight (BW) which did not vary more than 5 % during the study period. A total of 319 eligible records were analysed using multiple linear regression. Variation factors such as ownership, breed, sex and neutered status, bedding location, temperament and feeding habits were then analysed individually using a non-parametric model. Two models result from this study, one excluding age (*r*^2^ 0·813) and a more accurate one which takes into consideration the age in years (*r*^2^ 0·816). The second model was assessed with the main variation factors and shows that: MER (kcal) = *k*_1_ × *k*_2_ × *k*_3_ × *k*_4_ × *k*_5_ × 128 × BW^0·740^ × age^−0·050^/d (*r*^2^ 0·836), with *k*_1_ the effect of the breed, *k*_2_ the effect of sex and neutered status, *k*_3_ the effect of bedding location, *k*_4_ the effect of temperament and *k*_5_ the effect of the type of feed. The resulting model is very similar to the recommendations made by the National Research Council (2006) but a greater accuracy was obtained using age raised to a negative power, as demonstrated in human nutrition.

An analysis of the current prevalence of obesity in pet dogs leads to the alarming observation that 20–40 % of occidental adult pet dogs are overweight^(^^1^^,^^2^^)^. Despite the multiplication of data available on the subject, the energy requirements of pet dogs are not fully understood. A recent meta-analysis identified a total of 102 publications on the subject^(^^3^^)^. However, most of the studies were performed in laboratory conditions on kennel dogs, which preclude an accurate prediction of pet dogs’ maintenance energy requirements (MER), as this population is not representative of the needs of household pets^(^^4^^)^. Most available data are therefore limited by the husbandry of the animals in the associated study. Each pet dog population also differs from another with respect to elements such as breed types, activity levels, proportion of neutered subjects and lifestyle, where energy requirements vary according to ambient temperature^(^^4^^–^^6^^)^. Consequently, data from a geographical area must be considered carefully before being used in another location. Finally, most studies concerning dog energy requirements have small sample populations. The meta-analysis conducted by Bermingham *et al.*^(^^3^^)^ reviewed a sample of 713 dogs out of twenty-nine publications which contained an average of only 24·6 dogs by study.

The objectives of this study were (a) to determine the allometric exponent that best fits the French pet dog sample for the calculation of metabolic weight, (b) to determine if the energy needs are a function of age raised to some power and, (c) to propose an allometric equation that more accurately reflects the energy requirements of French pet dogs.

## Materials and methods

### Data collection

Participating students from the École Nationale Vétérinaire de Toulouse, France, were asked to follow an adult dog (≥1 year old, healthy, owned and fed the same way for 2 months) from their entourage. The student had to be either the dog's owner or a close relative or friend of the owner. The student had to accurately collect a detailed description of the pet and its feeding habits, with recordings of all ingested food, and an assessment of the body condition score (BCS, on a scale of 9)^(^^7^^)^. Data were collected over a period of more than 10 years as an exercise of carnivore nutrition teaching. To be included in the study, a reliable description of the nutritional composition had to be available and the quantity ingested accurately recorded. The composition had to be documented directly from the food packages and collected as part of the file. The amounts of food distributed at each meal or as snacks had to be weighed using an electric scale every day for several weeks, and controlled by the rate of consumption of a bag or a pack. Dogs were weighed weekly by the students and the dog's weight had to remain stable, i.e. not vary by more than 5 % for a period of at least 1 month. The BCS had to remain the same, i.e. at 4 or 5 on a scale of 9, as selected dogs were at their ideal body weight (BW). The temperament of dogs was subjectively assessed by the students. The metabolisable energy content of food was calculated with National Research Council 1974 and 1985 factors for homemade food and complete commercial food, respectively.

### Statistical methods

All the forms and nutritional tables from the dogs’ diets have been gathered in a unique spreadsheet in order to be processed using statistical analysis methods. The statistical analysis has been performed using R Core Team software and R Commander^(^^8^^,^^9^^)^ (version 3.2.2). The allometric equation aBWb was used to determine the relationship between the energy for maintenance and the ideal BW (in kg). Multiple regression analysis was used to examine the independent effects of (ideal) BW and also of BW and age on the energy requirements (kcal). Regarding allometric exponents, all measurements were analysed after natural logarithm transformation. The individual effects of all other factors taken into consideration in the study (ownership, breed, sex, neutering status, bedding location, temperament, type of diet and diet quality) were assessed with Kruskal–Wallis *H* tests followed by the Wilcoxon signed-rank test for all factors with more than two distinct levels.

### Exclusion criteria

All dogs with a diagnosed disease or with a weight varying by more than 5 % or with an ideal weight differing from actual weight have been removed from the study. All dogs aged less than 1 year, dogs working more than 1 h per d and dogs living in cages or in kennels were also excluded.

## Results

### Sample description

A total of 319 dogs of 4·91 (sd 3·49) years old and of seventy-eight different breeds formed the study population. The ten most represented breeds were Labrador retriever (*n* 25), Border collie (*n* 14), Belgian shepherd dog (*n* 14), golden retriever (*n* 13), German shepherd dog (*n* 12), fox terrier (*n* 11), Yorkshire terrier (*n* 10), Brittany spaniel (*n* 9), Beauce shepherd dog (*n* 9) and Pyrenean shepherd dog (*n* 8). The sex ratio was 0·87, with a slight majority of males (*n* 171) compared with females (*n* 148). Of the dogs, 24·3 % of the males and 55·4 % of the females were neutered. Dogs were owned by the student performing the study (*n* 49), by parents of the student (*n* 110), by another member of the student's family (*n* 23), by another veterinary student (*n* 17), with the remainder (*n* 25) not falling into the previous categories. The owner was not clearly identified for *n* 95 subjects. Most of the dogs spent the night inside the owner's house (*n* 192), *v*. *n* 42 that lived exclusively outside (night and day). For the remaining dogs (*n* 85), the living location was not clearly identified. With respect to behaviour, dogs were mostly considered as normal (*n* 138), as active (*n* 112), and more rarely as calm (*n* 58). Behaviour of dogs was subjectively assessed by the student and compared with the score done by the owner. Eleven dogs were excluded as they were not clearly identified as belonging to one of these categories. As for diet, the majority of the dogs were fed with a unique commercial dry food (*n* 274), but some of them consumed commercial moist food (*n* 13), a mix of at least two commercial products (*n* 11), a mix of a commercial product and leftovers (*n* 15) or a homemade diet composed of products destined exclusively for human consumption (*n* 6). Almost all of the dogs were fed once (*n* 149) or twice (*n* 147) per d, with a minority of subjects fed *ad libitum* (*n* 16), or three (*n* 6) or four times per d (*n* 1).

### Energy requirements for maintenance

Overall, mean BW was 23·18 (sd 13·55) kg (ranging from 1·1 to 80·0 kg). BW and MER were defined with MER = 119 × BW^0·732^/d (*r*^2^ 0·813), as a function of the dog BW (in kg), or MER = 128 × BW^0·730^ × age^−0·050^/d (*r*^2^ 0·816), as a function of BW and age (in years). As this second model allowed for a better correlation, energy requirements were expressed in kcal × kg^0·730^ × year^−0·050^/d further in the study.

### Individual effects of ownership, breed, sex and neuter status, husbandry and activity

There was no perceived effect of ownership on MER of dogs, with similar requirements in dogs belonging to students and others (Table 1, *P* = 0·42).

**Table 1.** Effect of owner, breed, sex and neutering status, bedding location, temperament and type of diet on maintenance energy requirements (in kcal × kg^0·730^ × year^−0·050^/d) of adult healthy pet dogs* (Mean values and standard deviations)

Factor	Level	*n*	Mean	sd	*P*
Full sample		319	128·0	42·2	
Owner	Veterinary student	66	141·8	58·6	NS
	Others	158	134·2	39·1	
Breed	Belgian and Beauce shepherd dogs	23	147·2^a^	33·4	<0·001
	Labrador and golden retrievers	38	119·4^b^	24·3	
	Others	258	135·3^c^	44·4	
Sex and neutered status	Entire females	37	139·3^a^	30·2	<0·01
	Entire males	56	131·2^ab^	25·6	
	Neutered females	46	118·9^b^	24·4	
	Neutered males	18	131·1^a,b^	40·0	
Bedding place	Outside	42	142·2^a^	24·6	<0·05
	Inside	192	135·3^b^	45·0	
Temperament	Normal and calm	196	132·8^a^	47·5	<0·05
	Active	112	138·8^b^	31·7	
Kind of food	Commercial dry complete	274	132·6^a^	28·5	<0·01
	Commercial moist complete	13	102·9^a^	36·2	
	Mix of commercial	11	121·5^a^	33·9	
	Commercial and leftovers	15	195·1^b^	121·4	
	Homemade diet	6	147·7^a^	86·5	

^a,b,c^ Mean values within a column (factor) with unlike superscript letters were significantly different.

* If there are missing data, the sum of dogs considered for each factor may be different from 319.

There was no difference in the MER (in kcal × kg^0·730^ × year^−0·050^/d) with respect to the breed for the ten most represented breeds in the study (considered without any grouping). Mean requirements for these breeds are shown in Table 2. However, significant differences appeared when groups were considered either for high (Belgian and Beauce shepherd dogs), low (Labrador and golden retriever) or average energy requirements (Border collie, German shepherd dog, fox terrier, Yorkshire terrier, Brittany spaniel, Pyrenean shepherd dog and others breeds) (Table 1). Some retrievers and shepherd dogs had a significantly different MER from other dogs (*P* < 0·001). Consequently, when considering this grouping, a correctional factor ranging from 0·88 for Labrador and golden retrievers to 1·09 for Belgian and Beauce shepherd dogs was justified.

**Table 2.** Maintenance energy requirements (in kcal × kg^0·730^ × year^−0·050^/d) of the ten main breed and other dogs (dogs belonging to other breeds but in low numbers) (Mean values and standard deviations)

Breed	*n*	Mean	sd
Labrador retriever	25	120·7	25·1
Border collie	14	135·6	23·3
Belgian shepherd dog	14	143·7	33·0
Golden retriever	13	116·8	23·5
German shepherd dog	12	139·5	19·5
Fox terrier	11	171·1	33·1
Yorkshire terrier	10	122·0	28·3
Brittany spaniel	9	126·9	32·3
Beauce shepherd dog	9	152·7	35·3
Pyrenean shepherd dog	8	136·9	33·4
Others	194	133·9	37·9

Surprisingly, only the MER for spayed bitches was significantly lower than that of the entire non-spayed female population (*P* < 0·01). This justifies a correcting factor of 0·85 on MER for neutered females.

Dogs that spent days and nights outside had a higher MER than dogs staying inside the owner's house at night (*P* < 0·05). This difference resulted in a correcting factor of 1·05 on MER for dogs sleeping outside.

There was no significant difference on the MER regarding the temperament of the dogs when comparing calm and normal. Dogs with active temperaments needed greater MER than the others (*P* < 0·05). This justified a correcting factor of 1·05 on MER of active dogs.

Concerning the type of diet, only dogs fed a mixture of commercial products and leftovers seemed to show a higher MER (*P* < 0·05) but the small size of this group (*n* 15) and the high standard deviation concerning such diet make the results inconclusive. Finally, there was no effect of meal frequency on MER.

Consequently, the MER may be calculated using the following equation:

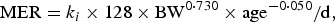

with *k*_*i*_, the correction factor for breed (0·88 for Labrador and golden retriever, 1·09 for Belgian and Beauce shepherd dogs), sex and neutered status (0·85 for spayed bitches), bedding location (1·05 for dogs sleeping outside) and temperament (1·05 for active dogs), respectively. However, the interactions between *k*_*i*_ could not be studied together, as inconstant variability of these prevented this study.

## Discussion

This study conducted on 319 healthy adult French pet dogs aimed to determine the MER as a function of ideal BW. Our results showed that MER is also a function of age, showing a slight decrease with time, as in humans. Thus, MER of these pet dogs can be expressed as a constant multiplied by the BW to the power 0·730 and multiplied by the age to the power −0·050. This also varies as a function of breed, neutered status, temperament and bedding location.

To the authors’ knowledge, this is the first MER study conducted on pet dogs in France. The sampling method, using the participation of veterinary students, allowed the constitution of a large sample with objective observations and avoided the use of veterinary clientele. Although it was conducted at the veterinary school based in Toulouse, students originate from every part of France, which increases the relevance of the conclusions at a national level. The students allowed us to conduct a feeding study in dogs for more than 1 month and only dogs with variation in BW of less than 5 %, free from disease, and having a good BCS, were included. Only dogs with a low activity level, i.e. less than 1 h/d of forced activity such as hunting or agility in addition to spontaneous exercise, were retained. In other words, working dogs were not considered in this study. Indeed, the time and intensity of work or training would have led to considerably higher energy requirements with a large disparity of MER.

Of the 579 records in the database, 260 were excluded because the eligibility criteria were not met or the data were incomplete (no BCS or ideal weight mentioned, unstable BW). The participation of veterinary students enabled the collection of a large sample. Students were trained prior to the study in order to reduce discrepancies in data collection methods. The recruitment of dogs may have been biased as the dogs of students and their relatives were probably better fed, more regularly walked and better monitored than the dogs of the general French population. However, no differences were observed between the ownership groups.

Our results show that metabolic weight for the sample population was equal to the BW raised to the power 0·730, which is similar to the results obtained by Brody *et al.* (1934)^(10)^, who proposed an allometric exponent of 0·73. However, since the use of the 0·75 allometric exponent proposed by Kleiber in 1961 for practical reasons, most authors calculate energy needs of dogs and express it in kcal × kg^0·75^/d, as suggested by the National Research Council^(^^4^^)^. It should be noted that the difference between 0·73 and 0·75 in the exponent leads to a difference of more than 7 % for a 30 kg animal. Furthermore, this allometric exponent is very different from 0·67 (2/3) used in cats or in species where mature and young animals are physiologically similar^(^^11^^)^, which might be true for all mammals, except dogs. Indeed, with increasing mature size, dogs become relatively taller at the shoulder and narrower at the hip^(^^12^^)^. The brain and several visceral organs including the liver and gastrointestinal tract become relatively smaller with increasing mature weight^(^^13^^)^.

In the proposed model, MER is proportional to the age raised to a negative power, in an allometric equation comparable with the one proposed in dogs^(^^5^^)^ and in humans^(^^14^^,^^15^^)^. Consequently ‘metabolic age’ can be defined as the age to the power of −0·050. Even if the exponent value seems to be insignificant, feeding a 5-year-old dog the same amount of energy as a 1-year-old dog with equal ideal BW can have significant consequences, such as excess weight or obesity. Otherwise, if the amount of energy dispensed is not decreased with age, this could lead to a weight gain of 5·6 or 7·7 kg per year, at 5 or 10 years, respectively, for a 30 kg dog.

Factors such as breed, sex and neuter status, husbandry and activity were studied with a non-parametric model because the inconstant variability of these factors prevented an assessment of possible interactions between the different factors. Although breed was responsible for a clear tendency in the variation on MER, inter-individual variability prevented a conclusive result. The significant variation in breeds resulted in a low headcount per breed. Consequently, several breeds were grouped, even breeds with a relatively ‘large’ headcount (*n* ≥ 8). Finally, three groups were considered, the retrievers group combining the Labradors and the golden retrievers, the shepherd dog group combining the Belgian shepherd dog and the Beauce shepherd dog and a final group, constituted of all remaining dogs in the study population. The results obtained should be considered carefully as it is likely that a number of breeds were not sufficiently represented in this sample to show significant difference with other dogs. Moreover, even if retrievers seem to have a lower and some shepherds a higher MER than average, this observation is difficult to extrapolate for all retrievers and shepherds. With respect to neutered dogs, the only significant difference shown by the study was for spayed bitches. Perhaps, the low headcount of castrated males could explain this result. This would have to be confirmed using a larger sample of dogs. Consequently this study cannot conclude if castrated males do or do not require a corrective factor when calculating their MER. The bedding location justifies a corrective factor for the dogs that spend day and night outside. This is to be considered with the variation of energy requirements as a function of ambient temperature^(^^4^^–^^6^^)^. This result only concerns metropolitan France and should be considered more carefully before extrapolation to other geographical regions. Moreover, breed is likely to be correlated with energy needs within different ambient temperatures, as it was observed in Pennsylvania by increasing the MER during winter for Labradors and beagles, but not for Siberian huskies^(^^5^^)^. Likewise, temperament, linked to spontaneous activity, has an influence on MER. Indeed, dogs with an active behaviour have significantly raised MER when compared with dogs with a normal or calm temperament. This experimental protocol did not take into account the leisure activity linked to sport or work because only pet dogs were considered.

In conclusion, the work of veterinary students allowed an estimation of MER of pet adult dogs. The allometric equation derived as a result of their work shows a significant correlation with previous estimates. In addition, the assessment of the individual pet dogs’ requirements could be improved if information on breed, sex, neutered status, bedding place and temperament is taken into account.
